# Silibinin Modulates NF‐κB Activation and Heat Shock Protein in Preeclampsia‐Like Rat Model

**DOI:** 10.1111/aji.70275

**Published:** 2026-07-03

**Authors:** C. F. Bannwart‐Castro, M. Romao‐Veiga, V. R. Ribeiro‐Vasques, M. T. S. Peracoli, J. C. Peracoli

**Affiliations:** ^1^ General and Applied Biology Post Graduate Program Institute of Biosciences Sao Paulo State University‐UNESP Botucatu Sao Paulo Brazil; ^2^ Experimental Research Unity ‐ UNIPEX Botucatu Medical School Sao Paulo State University‐UNESP Botucatu Sao Paulo Brazil; ^3^ Department of Gynecology and Obstetrics Botucatu Medical School Sao Paulo State University‐UNESP Botucatu Sao Paulo Brazil; ^4^ Department of Genetics Microbiology and Immunology Institute of Biosciences Sao Paulo State University‐UNESP Botucatu Sao Paulo Brazil

**Keywords:** Hsp70, NF‐κB, L‐NAME, preeclampsia, silibinin, cytokines

## Abstract

**Problem:**

Preeclampsia (PE) is a serious human pregnancy complication and remains the most complex hypertensive disorder due to its multifactorial nature and multisystemic effects. Despite the incomplete understanding of the PE pathophysiology, it is well‐accepted that placental changes in PE are associated with oxidative stress, increasing the excessive production of cytokines and other inflammatory mediators. Thus, agents such as silibinin, a non‐toxic natural polyphenolic flavonoid with antioxidant, anti‐inflammatory, and hepatoprotective properties could modulate this profile for better disease resolution. This study aimed to investigate the effect of silibinin treatment on Nω‐nitro‐L‐arginine methyl ester (L‐NAME)‐induced PE in rats, exploring its potential as a preventive anti‐inflammatory agent.

**Methods of Study:**

Pregnant Wistar rats were treated or not during gestation (days 10–19) with L‐NAME (70–80 mg/kg/day) in drinking water and with silibinin (100 mg/kg/day, orally) starting on days 0, 7 or 14 of pregnancy. Systolic blood pressure was recorded on gestation days 0 and 20. The rats were euthanized on day 20. Then, we evaluated proteinuria, maternal weight gain, and litter weight. Tumor necrosis factor‐ alpha (TNF‐α), interleukin‐1 (IL‐1β), IL‐6, IL‐10, interferon‐gamma (IFN‐γ), heat shock protein 70 (Hsp70) and NF‐κB activity were determined in liver and placenta homogenates.

**Results:**

Our findings indicated that silibinin treatment decreased the production of the inflammatory cytokines TNF‐α, IL‐1β, IFN‐γ, and reduced Hsp70 levels in the placenta and liver homogenate. NF‐κB activation was also decreased in these organs of silibinin‐treated groups, especially in the LN+SB0 and LN+SB7 groups.

**Conclusion:**

This study introduces a novel use of silibinin as a preventive agent in a PE‐like model that reproduces essential features of human PE, providing physiological relevance to the findings. Overall, our data emphasize the translational potential of silibinin for PE prevention, showing promising effects on key inflammatory and stress‐related pathways.

## 1 Introduction

Preeclampsia (PE) is an important obstetric disorder that affects previously normotensive women and complicates between 2 % and 8 % of all pregnancies, being the main cause of maternal and perinatal morbidity and mortality [1]. The disease is characterized by the onset of hypertension associated or not with proteinuria and another maternal target organ dysfunction after 20 weeks of gestation [2, 3]. Despite the incomplete understanding of the PE pathophysiology, it may be associated with abnormal placentation function, excessive maternal systemic inflammation, and endothelial dysfunction [4, 5, 6, 7]. Endothelial homeostasis in pregnancy is maintained by the endothelial nitric oxide synthase (eNOS) enzyme and nitric oxide well function. A decrease in eNOS concentration is observed in PE [8]. According to Sakowicz et al. [9], systemic oxidative stress and endothelial dysfunction in PE are triggered by abnormal development of the placenta, resulting in severe symptoms like hypertension and proteinuria.

Exacerbated systemic inflammation in PE is characterized by high production of pro‐inflammatory cytokines, such as interleukin‐1 beta (IL‐1β), IL‐6, and tumor necrosis factor‐alpha (TNF‐α), increased plasma concentration of heat shock protein 70 (Hsp70), and High mobility group Box 1 (HMGB1) [10, 11, 12, 13].

Pro‐inflammatory cytokines, chemokines, and molecules mediators of inflammation production depend on the activation of signal transduction via nuclear transcription factor‐kappa B (NF‐κB) [14]. NF‐κB regulates multiple aspects of innate and adaptive immune functions and represents a pivotal mediator of inflammatory responses by regulating the survival, activation, and differentiation of innate immune cells and inflammatory T cells [15]. The most common NF‐κB dimer (p50/p65) is present in an inactive form bound to inhibitory kappa B IIkB) in the cytoplasm. After stimuli with microbial components that bind to Toll‐like receptors, result in IkB degradation leading to NF‐κB translocation to the nucleus and initiation of transcription of genes associated with inflammatory response [15, 16].

Animal models are valuable tools for investigating PE pathogenesis, diagnostic criteria, and treatment methods. In the literature, several studies have employed Nϖ‐nitro‐L‐arginine methyl ester (L‐NAME), an inhibitor of eNOS, to induce a PE‐like model in rats [17, 18]. This animal is commonly accepted as a PE‐like model that mimics vascular pathology to better understand PE onset and progression.

Several products, such as Tetramethylpyrazine [19], Apocynin [20], Metformin [21], aspirin supplemented with quercetin [22], and Kefir peptides (KPs) derived from various prebiotic fermentations in whole milk [23], have been demonstrated to attenuate the consequences of NO inhibition in pregnant rats. These products show promise by alleviating hypertension, maintaining normal blood pressure levels until delivery, and exerting anti‐inflammatory and antioxidant effects [24].

Adjuvant treatments with natural products are constantly studied as targets for this disease. Silibinin (SB) is a chemically defined polyphenolic flavonoid and the main biologically active component of silymarin, a complex extracted from the seeds of milk thistle, *Silybum marianum* (Asteraceae; Flavolignane), that exhibits potent hepatoprotective, antioxidant, and anti‐inflammatory activities [25, 26]. Silibinin's pharmacokinetic issues and safety are well‐known due to its long use for treating liver disease [27, 28] and has been pointed out as an alternative secure non‐toxic PE treatment in vitro and in vivo [14, 17, 29, 30, 31].

The SB anti‐inflammatory and antioxidant effects are continually described in the literature and attributed to suppressing NF‐κB‐regulated gene products [32, 33, 34]. This role suggests that NF‐κB pathway inhibitors might be effective targets for the treatment of chronic inflammatory diseases [25]. Since PE is associated with an intense inflammatory response, and the L‐NAME‐induced PE‐like rat model may be a promising means of investigating the pathogenesis of PE and evaluating therapies for this condition [18], the objective of this study was to determine if the SB treatment in PE‐like model in rats could modulate the production of inflammatory cytokines, heat shock proteins production, and NF‐κB activation in target organs like liver and placenta.

## 2 Material and Methods

### 2.1 Animals

Male and nulliparous female Wistar‐Kyoto rats (aged 10–12 weeks) weighing between 220 and 260 g were purchased from the Central Animal House Services (CEMIB) at The University of Campinas (UNICAMP). The animals were maintained under controlled temperature (22±2°C) and lighting (12 h light/dark cycle), and with free access to food and water. The protocols for animal use and procedures for the experiments described here were approved by the Animal Ethics Committee of the Institution, and performed following the ethical guidelines established by the Brazilian College for Animal Experimentation (COBEA).

### 2.2 Model of PE‐Induced in the Pregnant Rats

A PE‐like model was established via oral administration of Nϖ‐nitro‐L‐arginine methyl ester (L‐NAME) to normal pregnant rats as has been described previously [17, 35]. The experimental sequence was divided into four periods: adaptation, mating, pregnancy, and treatment. Before the experiment, the animals were acclimated to laboratory conditions for 7 days. After mating overnight with adult males, the presence of sperm in vaginal smears confirmed pregnancy on day 0. After pregnancy confirmation, each rat was weighed and placed in an individual metabolic cage. Pregnant Wistar rats were treated or not, with L‐NAME (70‐80 mg/kg/day) in drinking water from day 10 to 20 of gestation. Before starting the L‐NAME treatment, the mean volume of liquid ingested by the pregnant rats in each cage was determined by measuring the volume of water that the rats had drunk. This calculated volume was then used to determine the amount of the drug to be diluted directly in the drinking water to give the desired dose per Kg of body weight per day.

### 2.3 Treatment Solution

Silibinin (SB) (Sigma‐Aldrich, Inc., St Louis, MO, USA) at a concentration of 100 mg/Kg/day was prepared as a suspension in 0.4% carboxymethylcellulose (CMC) (Sigma‐Aldrich) administered by gavage (intragastric route). The concentration employed was previously standardized in a pilot study with Wistar pregnant rats, showing that the dose did not affect the reproductive outcome.

### 2.4 Experimental Groups

**FIGURE 1:**
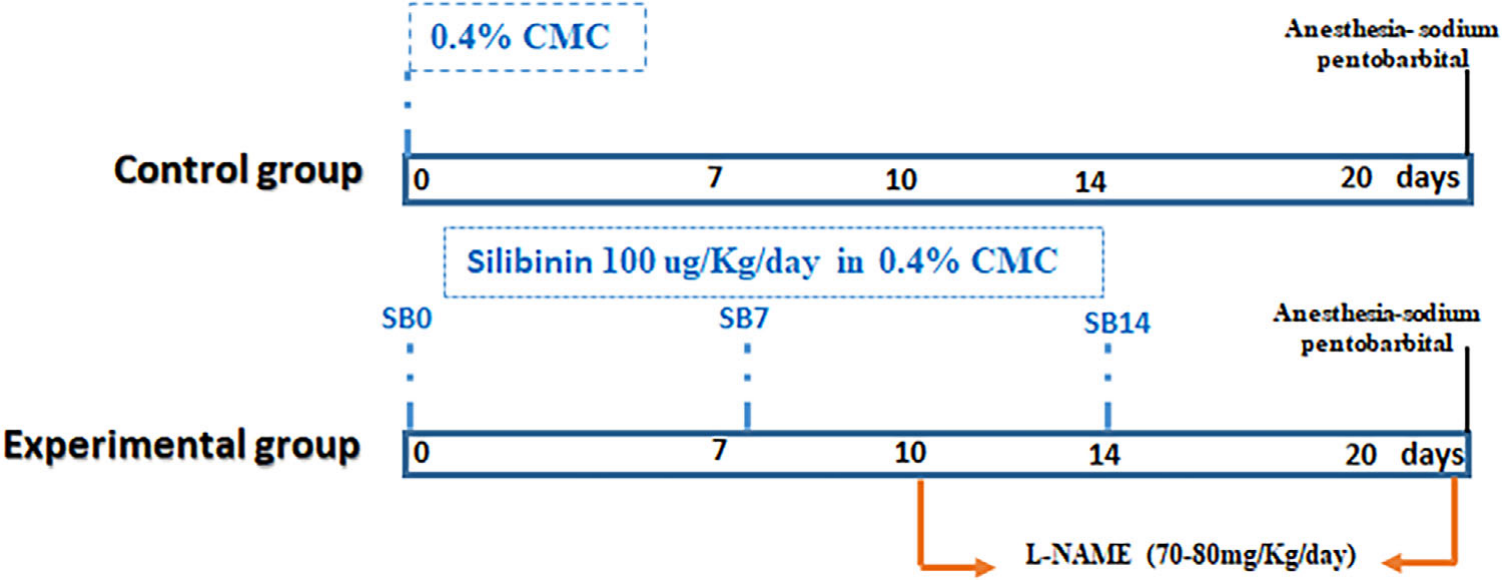
Experimental design: Wistar rats (*n* = 6) in each group treated with silibinin from days 0, 7, or 14 of pregnancy, and with L‐NAME from day 10 to day 20. Abbreviations: L‐NAME = Nω‐nitro‐L‐arginine methyl ester; SB = silibinin; CMC = carboxymethylcellulose.

As demonstrated in Figure 1, pregnant rats were separated into five groups (*n* = 6 animals in each group). Groups: 1. Control (normotensive group treated only with a 0.4% carboxymethylcellulose (CMC‐silibinin vehicle) by gavage since day 0 to mimic the stress of handling the animal to perform gavage); 2. L‐NAME (rats treated with L‐NAME from 10 up to 20 days of gestation); 3. LN+SB0 (animals treated with SB since day 0); 4. LN+SB7 (animals treated with SB since day 7); 5. LN+SB14 (animals treated with SB since day 14). The L‐NAME, LN+SB0, LN+SB7, and LN+SB14 groups received L‐NAME in drinking water from day 10 and all treatment lasted until day 20. The animal groups were subjected to blood pressure measurements on days 0 and 20 of pregnancy. Proteinuria, maternal weight gain, litter weight, cytokines, Hsp70, and p65 NF‐kB in liver and placental homogenates was evaluated on day 20 of pregnancy.

### 2.5 Blood Pressure and Proteinuria Measurement

Blood pressure was measured on days 0 and 20 of pregnancy by the non‐invasive tail‐cuff method employing a pressure meter (Panlab, LE 5002, Barcelona, Spain) after animals were pre‐warmed for 10 min at 37°C in a heating chamber. Blood pressure was obtained five times, and the mean value of each rat was recorded. On day 20 of pregnancy, 24 h urine was collected from each animal and stored at −80°C for urinary protein determination by Lowry's method [36]. The method's principle is based on a mixture of molybdate to tungstate and phosphoric acid (Folin‐Ciocalteau), which is reduced when it reacts with proteins in the presence of catalytic copper (II) and produces a compound with an absorption maximum at 750 nm.

### 2.6 Laparotomy, Samples Collection and Homogenates of Liver and Placenta Preparation

On day 20 of pregnancy, the rats were submitted to anesthesia with sodium pentobarbital (50 mg/kg of body weight, i.p.) and placed in the supine position for the cesarean section, with abdominal incision opening through the medial incision of the skin, abdominal musculature, and peritoneum. Maternal weight gain and litter weight were recorded. Liver and placenta were obtained from the female rats during cesarean section. To prepare the homogenates, liver and placental tissues were washed four times in ice‐cold phosphate‐buffered saline (PBS) to remove the remaining blood. Each rat's liver and placenta segments were weighed and homogenized in 10 mL of ice‐cold PBS and protease inhibitors (complete protease inhibitors cocktail; Sigma‐Aldrich, St. Louis, MO, USA). The tissues were fully homogenized with a Powergen homogenizer (Fisher Scientific, Pittsburg, PA, USA) for 30 s on ice. Homogenates were centrifuged at 12000 g for 20 min at 4°C. The supernatant from liver and placenta homogenates was collected and filtered through a 0.22 µm millipore membrane, and aliquots were stored at –80°C until the analysis.

### 2.7 Cytokines and Hsp70 Determination

TNF‐α (Catalog MTA00B), IL‐1β (Catalog DY401), IFN‐γ (Catalog MIF00), IL‐6 (Catalog DY406), IL‐10 (Catalog M1000B), and Hsp70 (Catalog DYC1663‐2) concentrations in liver and placenta homogenates were determined by enzyme‐linked immunosorbent assay (ELISA) employing Quantikine ELISA kits (R&D Systems, Minneapolis, MN, USA), according to the manufacturer's instructions. The concentration of cytokines detected in the liver and placenta was adjusted to g of tissue per mL and represented as pg/g of tissue.

### 2.8 p65 NF‐κB Determination

Liver and placental homogenates from each group were employed to determine the levels of p65 NF‐κB using a transcription factor Assay Kit (Catalog ab324202, Cayman Chemical, Thermo Fisher Scientific, Inc., USA) according to the manufacturer's instructions. Total protein concentrations were determined by Lowry's method [36], and p65 NF‐κB was expressed as µg/µg of protein in tissue.

### 2.9 Statistical Analysis

The results were expressed as mean ± standard deviation (SD). All data were analyzed by one‐way ANOVA and/or the Tukey‐Kramer Multiple Comparisons test, using GraphPad Prism version 6.01 (GraphPad, CA, USA). Statistical significance was adopted at *p* < 0.05.

## 3 Results

### 3.1 Characteristics of Pregnant Rats and Laboratory Parameters on Day 20 of Pregnancy

Compared with the control group (Co) and groups treated with SB (SB0, SB7, and SB14) the maternal weight gain of the L‐NAME group was significantly lower, while proteinuria was significantly higher. Results for litter weight showed significant differences among L‐NAME and control (Co), LN+SB0, and LN+SB7 groups. No significant differences were detected between L‐NAME and LN+SB14 (Table 1).

**TABLE 1:** Characteristics of pregnant rats and laboratory parameters.

Characteristics	Groups (*n* = 6)				
	Co	L‐NAME	LN+SB0	LN+SB7	LN+SB14
Maternal weight gain (g)	61.5 ± 10.4	30.5 ± 9.2^*^	55.4 ± 9.7	65.6 ± 10.9	50.2 ± 11.3
Litter weight (g)	57.6 ± 8.2	48.8 ± 7.1^+^	56.3 ± 6.9	69.9 ± 8.8	49.7 ± 9.4
Proteinuria (mg/dL)	17.8 ± 3.2	84.2 ± 15.7^*^	22.3 ± 3.7	17.2 ± 2.2	35.7 ± 8.9

*Note:* Data are expressed as the mean ± SD.

* *p*<0.05 vs Co. LN+SB0. LN+SB7 and LN+SB14.

^+^ *p*<0.05 vs Co. LN+SB0. LN+SB7.

### 3.2 Silibinin Reduces Systolic Blood Pressure of PE‐Like Rats

**FIGURE 2:**
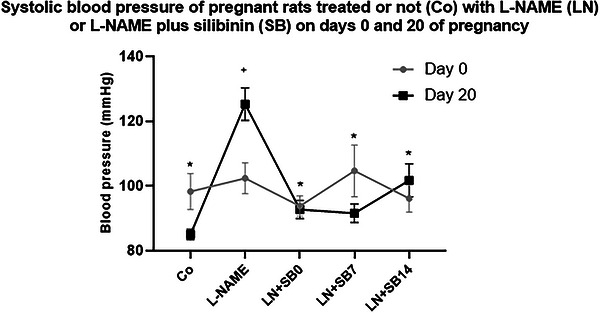
Systolic blood pressure of pregnant rats treated or not (Co) with L‐NAME or L‐NAME plus silibinin (SB) on days 0 and 20 of pregnancy. Blood pressure was recorded non‐invasively using the tail‐cuff method. Data are expressed in millimeters of mercury (mm Hg) and presented as mean ± SD. * (*p*<0.05) versus L‐NAME; + *p* <0.05 versus Co and LN‐SB0.

Figure 2 shows the results of systolic blood pressure (SBP) evaluated on days 0 and 20 of pregnancy. No significant differences were detected between groups on day 0 of pregnancy before animals’ treatment with L‐NAME or SB (Figure 2). However, on day 20 of pregnancy, higher levels of SBP were observed in animals treated with L‐NAME (Figure 2) compared with animals of the control group, not treated with L‐NAME, groups treated with SB, and also animals on day 0 of pregnancy.

### 3.3 Silibinin Modulates Inflammatory Cytokines Production of PE‐Like Rats

#### 3.3.1 Cytokine Profile Levels in Liver Homogenate

**FIGURE 3:**
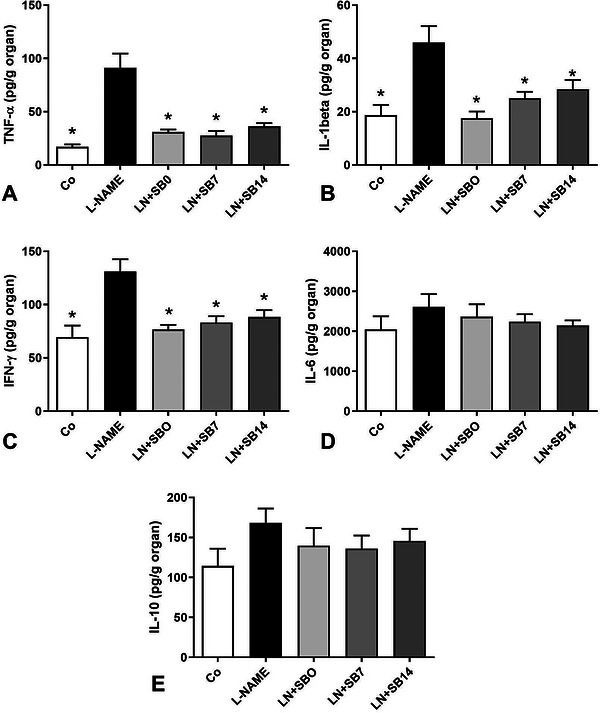
The concentration of TNF‐α (A), IL‐1β (B), IFN‐γ (C), IL‐6 (D), and IL‐10 (E) in liver homogenates of pregnant rats (*n* = 6) treated or not with L‐NAME or with L‐NAME+silibinin (SB) in different periods of pregnancy. ⁎ (*p*<0.05) versus L‐NAME (ANOVA).

Figure 3 shows that the L‐NAME group produced higher levels of TNF‐ɑ (Figure 3A), IL‐1β (Figure 3B), and IFN‐γ (Figure 3C) than the control group. The treatment with SB significantly reduced the production of these cytokines in the LN+SB0, LN+SB7, and LN+SB14 groups compared with the L‐NAME group. Production of IL‐6 (Figure 3D) and IL‐10 (Figure 3E) showed no significant difference between all the groups studied.

#### 3.3.2 Cytokine Profile Levels in Placental Homogenate

**FIGURE 4:**
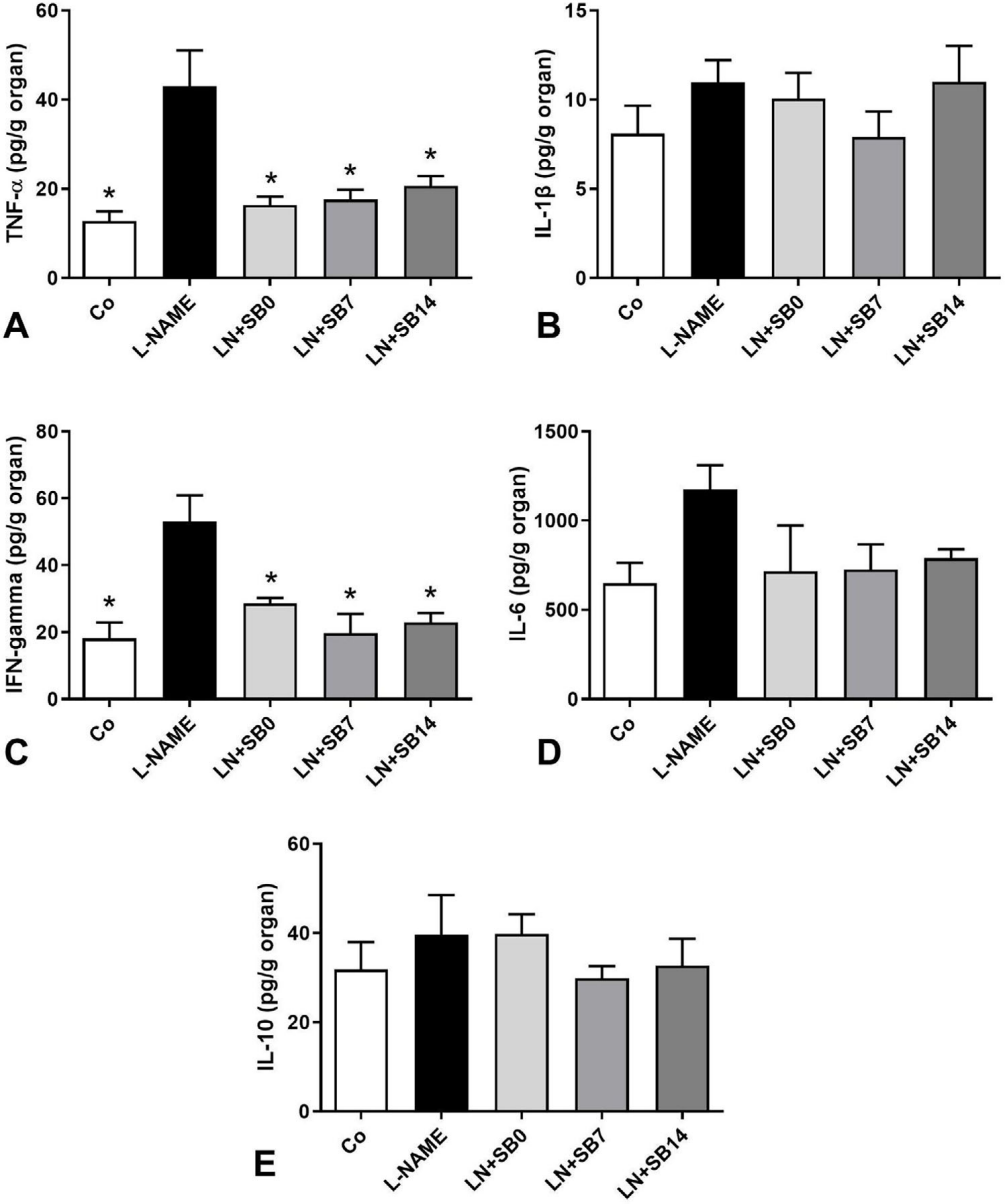
The concentration of TNF‐α (A), IL‐1β (B), IFN‐γ (C), IL‐6 (D), and IL‐10 (E) in placental homogenates of pregnant rats (*n* = 6) treated or not with L‐NAME or L‐NAME+ silibinin (SB) in different periods of pregnancy. ⁎ (*p*<0.05) vs. L‐NAME (ANOVA).

The production of TNF‐ɑ (Figure 4A), and IFN‐γ (Figure 4C) in the placenta was significantly higher in the L‐NAME group when compared to the control group. The treatment with SB led to a significant decrease in the production of these cytokines by the placenta of groups LN+SB0, LN+SB7, and LN+SB14. The results show that there were no significant differences in the production of IL‐1β (Figure 4B), IL‐6 (Figure 4D), and IL‐10 (Figure 4E) in the placenta among the groups.

### 3.4 Silibinin Mitigates Hsp70 Concentration Detected in the Liver and Placenta Homogenates of PE‐Like Rats

**FIGURE 5:**
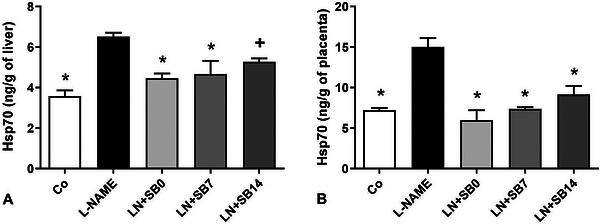
The concentration of Hsp70 detected in liver (A) and placenta (B) homogenates of pregnant rats (*n* = 6) treated or not with L‐NAME or L‐NAME + silibinin (SB) in different periods of pregnancy. ⁎ (*p*<0.05) versus L‐NAME; + (*p*<0.05) versus Co (ANOVA).

Compared with Co, LN+SB0, and LN+SB7 groups, the animal treatment with L‐NAME induced a significant increase in the levels of Hsp70 in the liver. No significant differences were detected between animals treated with L‐NAME and animals treated with LN+SB14. In the group LN+SB14, the levels of Hsp70 in liver homogenate were significantly higher than in the Co group. (Figure 5A). The analysis of the placenta homogenates showed significantly higher levels of Hsp70 in the animals treated only with L‐NAME compared with the other groups Co, LN+SB0, LN+SB7, and LN+SB14. (Figure 5B).

### 3.5 Silibinin Mitigates NF‐кB Activity in the Liver and Placenta of PE‐Like Rats

**FIGURE 6:**
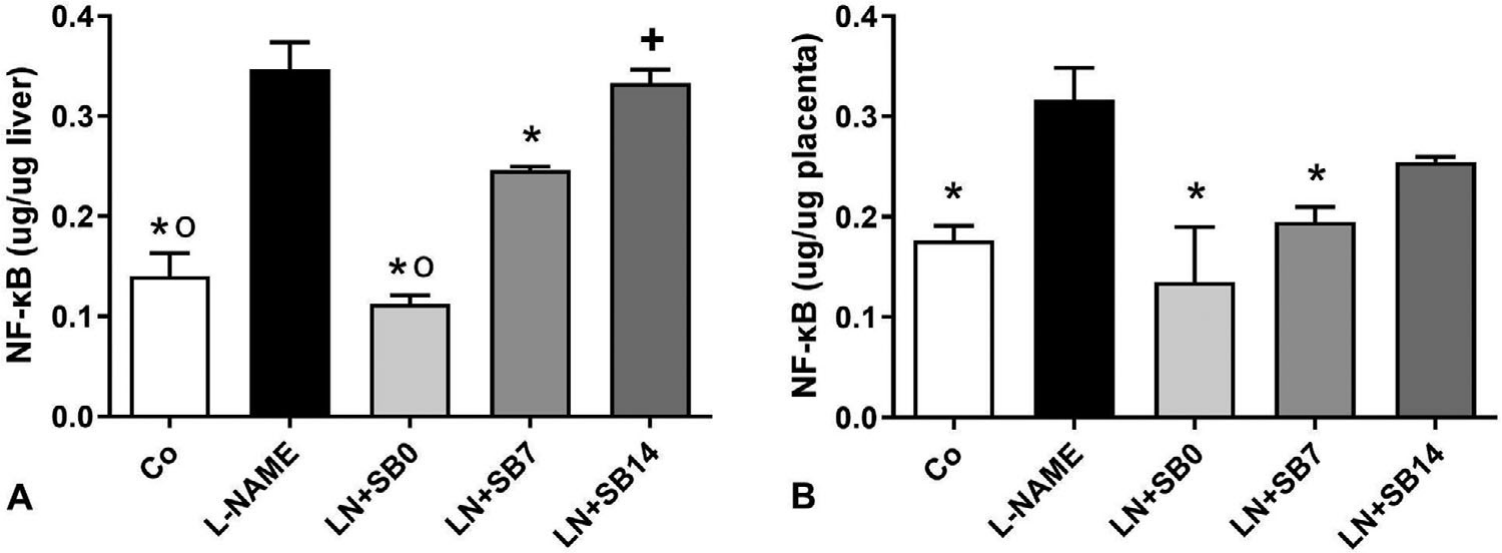
The concentration of p65NF‐кB detected in the liver (A) and placental homogenates (B) from pregnant rats (*n* = 6) treated or not with L‐NAME and/or L‐NAME + silibinin (SB). * (*p*<0.05) versus L‐NAME; • (*p*<0.05) versus LN+SB7; + *p*<0.05 vs Co, LN+SB0, LN+SB7 (ANOVA).

The L‐NAME group shows a significantly higher activity of NF‐кB in the liver (Figure 6A) compared with the control group (Co), LN+SB0, and LN+SB7 groups treated with SB. No significant differences were detected between pregnant rats treated with L‐NAME and rats treated with LN+SB14. In the group LN+SB14, the levels of NFkB in the liver homogenate were significantly higher than in the Co, LN+SB0, and LN+SB7 groups. The levels of NFkB in the control and LN+SB0 groups were significantly lower than in the LN+SB7 group.

In placental homogenate, the determination of NF‐кB showed significantly higher levels in the L‐NAME group compared with control (Co), LN+SB0, and LN+SB7 groups. No significant differences were detected between pregnant rats treated with L‐NAME and rats treated with LN+SB14 (Fig 6B).

## 4 Discussion

The present study demonstrated that silibinin's treatment in a PE‐like model induced by L‐NAME in pregnant rats reduced blood pressure and proteinuria compared with animals without treatment. SB showed its anti‐inflammatory properties by reducing the production of pro‐inflammatory cytokines, and Hsp70 levels, and by reducing NF‐κB activation observed in liver and placental homogenates from rats. The results showed that on day 20 of pregnancy, systolic blood pressure and proteinuria were significantly lower in all SB‐treated groups than those treated only with L‐NAME, suggesting that SB administration decreased blood pressure and 24‐h proteinuria. Additionally, animals treated with SB maintained maternal weight gain compared to those treated only with L‐NAME. L‐NAME treatment resulted in lower litter weight in the L‐NAME group than in the control and LN+SB0 and LN+SB7 groups, indicating a harmful effect of L‐NAME on litter weight. Inhibition of nitric oxide synthase by L‐NAME causes vasoconstriction and decreased uteroplacental blood flow, leading to reduced fetal weight [18].

These experimental animals have significant anatomical and behavioral advantages over mice for evaluation of PE pathogenesis, diagnosis, and treatment [37]. The PE‐like condition induced by L‐NAME in rats is a good model for studying hypoxia, nitric oxide dysregulation, hypertension, angiogenesis disturbances, and unbalanced maternal immune responses [38]. Many recent studies employed the L‐NAME model in pregnant rats to evaluate placental physiology [38], and anti‐hypertensive and anti‐inflammatory drugs such as aspirin [22]; pyrroloquinoline quinone [39], and natural products obtained from plants like vinifera grape skins extracts [35] and Apocyanin [20]. A previous study of our group employing this PE‐like model and Silibinin (SB) treatment showed that this flavonoid protected against the deleterious effects of L‐NAME and improved reproductive outcomes. The animals treated with SB showed a reduction in blood pressure and proteinuria, normalization of platelet numbers, an increase in the number of live fetuses, a reduction in the percentage of post‐implantation loss, and recovery from liver injury [17].

In our present study, SB treatment decresed TNF‐α, IL‐1β, and IFN‐γ production in the liver of all groups treated with L‐NAME. SB treatment also reversed the higher levels of TNF and IFN‐γ detected in placental homogenates from all treated groups. These findings indicate that SB plays an anti‐inflammatory role by downregulating pro‐inflammatory cytokine production, an important factor in PE pathogenesis. In a previous study of experimental PE induced by L‐NAME in rats, we demonstrated an association between high levels of TNF‐α, IL‐1β, and IFN‐γ in placental homogenate and an intense mononuclear cell infiltrate in the liver parenchyma, especially in the periportal space. The treatment with SB reduces this cellular infiltrate and the pro‐inflammatory cytokine levels [17]. The anti‐inflammatory activity of SB has been attributed to its ability to downregulate the NF‐κB pathway, mainly involved in the synthesis of these pro‐inflammatory cytokines [22, 23, 39], alleviating the inflammatory profile in the PE‐like model and might explain the significant effects of SB on TNF‐α, IL‐1β, and IFN‐γ levels detected in liver and placental homogenates in our results. In pregnant women with PE previous studies in vitro showed the downregulatory effect of SB on the inflammatory cytokines TNF‐α, IL‐1β, IL‐6, IL‐12, IL‐17A produced by peripheral blood cells of innate and adaptive immunity [30, 31, 34]. Moreover, SB showed an immunomodulatory effect on the NLRP3 inflammasome activation in monocytes from preeclamptic women [14] confirming its role in the attempt to control the systemic inflammation of PE.

In this present study, we evaluated SB's effects on other inflammatory markers in the liver and placenta of PE‐like animals. The higher levels of Hsp70, detected in placental homogenates of animals treated with L‐NAME were decreased after SB treatment in all groups LN+SB0, LN+SB7, and LN+SB14. The results of liver homogenate show lower levels in LN+SB0 and LN+SB7 groups. To our knowledge, this is the first study that shows the modulatory effect of this flavonoid on Hsp70 production in PE‐like rats. SB seems to decrease Hsp70 production in the liver and placenta of animals treated with L‐NAME. The stress environment induced by L‐NAME appears to increase Hsp70 levels in these organs. Hsp70 may act as a molecular chaperone that could be produced in response to a physiological or environmental effect of stress, such as elevation in temperature, or oxidative stress [40, 41]. According to some authors [12, 42, 43], a higher concentration of Hsp70 found in plasma or serum from preeclamptic women may indicate systemic inflammatory response, oxidative stress, and hepatocellular injury. Evidence from our previous study demonstrated that plasma Hsp70 levels were significantly higher in early‐onset PE women compared to late‐onset ones, suggesting its role in severe cases of PE [10].

Extracellular Hsp70 functions as an intercellular stress signaling molecule, acting as a danger signal called damage‐associated molecular patterns (DAMPs), indicative of nonphysiological conditions, such as cellular stress or damage, that trigger innate and adaptive proinflammatory immune responses [40]. Another important source of Hsp70 would be the innate immunity cells such as monocytes which are systemically activated in PE and produce both inflammatory cytokines and reactive oxygen intermediates [10, 44]. Romao‐Veiga [12] showed that monocytes from preeclamptic women stimulated with Hsp70 released higher levels of TNF‐α and IL‐1β, suggesting the participation of this DAMP in the systemic inflammatory response that characterizes PE.

Placental ischemia, oxidative stress, and maternal systemic inflammation, which are the major elements in the pathogenesis of PE have been shown to induce the expression of Hsp70. A systematic review and meta‐analysis conducted by Saghafi et al. [45] demonstrated the association between higher serum Hsp70 levels and PE. The expression of Hsp70 and eNOS is highly increased in placental tissue from PE women compared with normotensive pregnant women and could be a response to severe endothelial dysfunction in placental villous tissue [46]. An excessively high Hsp70 level could be responsible for increased expression of molecules related to MHC‐I (MICA and MICB) [47]. These molecules interact with the activating NKG2D receptor in NK cells, the main immune cell in the decidua, augmenting cytotoxicity and pro‐inflammatory cytokine release in this microenvironment [48]. Thus, our results suggest that SB can attenuate Hsp70 levels detected in placental homogenates of PE‐like rats, and might indirectly contribute to attenuating the inflammatory state promoted by higher levels of Hsp70 and NK cytotoxicity via the MICA‐NKG2D axis.

In our results, we detected high NF‐κB activity in placental and liver tissues of the L‐NAME‐induced PE‐like group. It seems that SB ameliorates this exaggerated inflammatory state in PE because both Hsp70 and p65 NF‐κB showed a significant decrease in concentration after treatment with SB. These results of SB downregulation of p65 NF‐κB activity in the liver and placental homogenate are in line with our previous results showing the effects of SB in inactivating TLR4/p65NF‐κB signaling in the culture of monocytes from pregnant women with PE [14].

Among the limitations of our study, the use of a controlled experimental model to simulate a heterogeneous disease as PE seems to be insufficient. However, this model has an advanced understanding of SB effects of alleviating key parameters of the disease. The early SB treatment from days 0 and 7 of pregnancy was important to mitigate the exacerbated inflammatory state detected in PE‐like rats, reducing pro‐inflammatory cytokines levels in homogenates of the liver and placenta. Here we also observe important and unpublished results of diminished NF‐κB activity in the liver and placental homogenates, such as a decrease in Hsp70 concentration in these key organs of PE damage.

## 5 Conclusions

Our findings suggest that silibinin could be a new treatment option and that the L‐NAME‐induced PE model is useful for investigating inflammation during pregnancy. The changes observed in placental and liver markers highlight the potential of silibinin in real‐world applications and indicate that this natural antioxidant and anti‐inflammatory flavonoid might help treat, prevent, and personalize care for PE. Future human studies are necessary to improve this adjuvant treatment. Future human studies are needed to improve this adjuvant treatment.

## Funding

This work was supported by the Sao Paulo Research Foundation‐ FAPESP, Brazil (Grant number 2010/00985‐9) and Coordenação de Aperfeiçoamento de Pessoal de Nível Superior‐ CAPES.

## Ethical statement

The study was approved by the Animal Experimentation Ethics Committee of the Botucatu Medical School—UNESP, under protocol number 590.

## Conflicts of Interest

The authors declare that they have no known competing financial interests or personal relationships that could have appeared to influence the work reported in this paper. All the authors declare they have no conflicts of interest.
